# To Check or Not to Check? A Comment on the Contemporary Psychometrics (ConPsy) Checklist for the Analysis of Questionnaire Items

**DOI:** 10.3390/ejihpe13100151

**Published:** 2023-10-06

**Authors:** Alexander Robitzsch

**Affiliations:** 1 IPN—Leibniz Institute for Science and Mathematics Education, Olshausenstraße 62, 24118 Kiel, Germany; robitzsch@leibniz-ipn.de; 2 Centre for International Student Assessment (ZIB), Olshausenstraße 62, 24118 Kiel, Germany

**Keywords:** psychometrics, factor analysis, assessment, measurement, checklist, scale, reliability, measurement invariance

## Abstract

In a recent paper, the first version of the contemporary psychometrics (ConPsy) checklist for assessing measurement tool quality has been published. This checklist aims to provide guidelines and references to researchers to assess measurement properties for newly developed measurement instruments. The ConPsy checklist recommends appropriate statistical methods for measurement instrument evaluation to guide researchers in instrument development and to support peer review. In this opinion article, I critically review some aspects of the checklist and question the usefulness of certain psychometric analyses in research practice.

## 1. Introduction

In a recent paper, researchers [1] published the first version of the contemporary psychometrics (ConPsy) checklist for assessing measurement tool quality (*PsyArXiv*; 15 August 2023; https://psyarxiv.com/t2pbj/, accessed on 30 August 2023). This checklist aims to provide guidelines and references to researchers to assess measurement properties for newly developed measurement instruments. The ConPsy checklist recommends appropriate statistical methods for measurement instrument evaluation to guide researchers in instrument development and to support peer review. ConPsy enables reviewers to evaluate the employed statistical methods in an article by a point system in a checklist format.

In this opinion article, I critically review some aspects of ConPsy recommendations. Notably, I would rather highlight the points of disagreement than emphasize the aspects of agreements. Readers might question the value of discussing a concrete checklist for psychometrics. It seems to me that the perspectives in the ConPsy checklist are typical for the field of psychometrics, and my critique applies to many textbooks in the field. I conclude my thoughts on whether there can be a universally applicable checklist with which a broad majority of researchers would agree.

The remainder of the paper is mainly structured according to the topics treated in the ConPsy checklist.

## 2. Sample Size Requirements

ConPsy discusses required sample sizes for latent variable models such as exploratory factor analysis (EFA) and confirmatory factor analysis (CFA). The ConPsy authors state that (see also [2,3])

“In the absence of detailed information about the model and data complexities, a common approach is to consider the subjects-to-variables (STV) ratio, which refers to the ratio of the number of participants (subjects) to the number of measured variables (items) in the study.”

“ConPsy recommends a minimum sample size of 200 individuals as a reasonable guideline for models with up to 25 items.”

“As a general guideline, ConPsy recommends a minimum STV ratio of 8:1. However, [...], smaller STVs (0.7) are acceptable in larger samples exceeding 300 individuals.”

I would like to acknowledge the fact that ConPsy also notes that required sample sizes in FA depend on several factors (e.g., number of items, estimation method, presence of missing data, model complexity). However, I think that a decision on whether an FA is appropriate for a given sample size should not rely on STV. In models with more items, the number of parameters will necessarily increase. Hence, the STV decreases and, according to ConPsy, FA would generally not be advised if the STV falls below a certain cutoff. However, for a fixed sample size, the amount of information in the data increases with the number of items. If model complexity is held constant, then having more items is generally not an issue. For example, the information about a latent factor variable *F* in a one-factor model increases if the number of items is increased. In this case, the number of parameters in the model would be increased, and the STV would decrease. However, the precision of estimated model parameters increases because *F* is more reliably estimated. This is in contrast to the statement that a low STV is critical. In fact, [4] demonstrated that the precision in estimated model parameters in factor models increases when the number of items per factor increases, although the STV is getting smaller.

I think that it is always much more appropriate to compute standard errors of estimated model parameters of interest (e.g., factor loadings, latent correlations, residual correlations, etc.). The standard errors provide a better insight into whether particular decisions made due to outcomes of the FA are reliable. For example, the value of a standardized factor loading of 0.40 could be a cutoff for deciding whether items should remain in the scale or not. Suppose that the estimated factor loading would be 0.20 or 0.30. I argue that an item should only be eliminated if the upper bound of a confidence interval (i.e., estimated loading plus two times its standard error) does not exceed the cutoff of 0.40. Otherwise, researchers can remove items from a scale just due to sampling errors. Of course, researchers should only make use of FA software that also reports standard errors for all estimated model parameters. Alternatively, researchers can apply resampling methods by themselves to assess the stability of estimated model parameters.

I also want to emphasize that stabilized parameter estimates in factor analysis can frequently be obtained by restricting measurement error variances to be positive to avoid Heywood cases. Applying constrained maximum likelihood or Bayesian estimation is particularly helpful in small samples [5].

It is always beneficial to conduct power analysis to determine a minimally required sample size before carrying out a study (i.e., administering the questionnaire to a sample of persons). There is a wide range of literature that can be consulted for power analysis [3,6,7].

It should be emphasized that the assessment of model fit in CFA by means of fit statistics is controversial because it is unlikely that cutoff values for fit statistics are generalizable for models of differing complexity [8,9]. Dynamic fit indices might be helpful in determining model-specific cutoff values for fit statistics [10,11,12].

Importantly, ConPsy highlights that the application of CFA after obtaining results from an EFA requires a part of the dataset not used in EFA:

“It is essential to consider that when both confirmatory factor analysis (CFA) and exploratory factor analysis (EFA) are employed in a study, the sample size requirement doubles, as each method necessitates separate datasets.”

I totally agree with this recommendation. EFA should be computed on a training sample dataset, while CFA should rely on a validation dataset. The full dataset could be split into two parts: one part for the EFA and the other part for the CFA. I think that this distinction is the most important part of Section “Sample size” in ConPsy, while everything regarding STV ratios can be mainly ignored.

## 3. Recurring Call for Ordinal Factor Analysis

In the social sciences, categorical rating scales with four or five points are frequently used. ConPsy has a clear opinion on how to treat such ordinal items adequately (see also [13]):

“It is unfortunately common in applied psychometrics to encounter situations where categorical data is mistakenly treated as continuous and normally distributed, particularly in the context of factor analysis. ConPsy emphasises the importance of using the appropriate method for each type of data at every stage of the analysis. It highlights the need to recognise and account for the categorical nature of the data to ensure accurate and valid results.”

This statement seems to imply that inaccurate results were obtained if FA based on the normal distribution (i.e., a continuous distribution) for ordinal items was applied. Later in the article, ConPsy explains their reasoning:

“ConPsy advises the use of factor extraction methods for categorical data when the items a) have fewer than five response options, b) have five options but floor and ceiling effects are present (common for instance when screening tools for a certain diagnosis are administered to general population), or if c) the data are ordinal, and the assumption of equidistant categories does not necessarily hold. Biased estimates may emerge when the number of categories is below five and/or the categorical distribution is highly asymmetric (Beauducel & Herzberg, 2006; Rhemtulla et al., 2012).”

First of all, I would like to emphasize that the statement “biased estimates emerge” is empirically meaningless for a concrete dataset. Bias in parameter estimates can only be assessed if researchers know the truth. That is, they assume a known data-generating model and wish to demonstrate that their method of choice outperforms other methods. Ref. [14] simulates ordinal items from an FA that holds for the matrix of polychoric correlations (i.e., one can write $\Sigma^{*}=\Lambda \Phi \Lambda^{⊤}+\Psi$ for the polychoric correlation matrix $\Sigma^{*}$). Hence, the FA model is correctly specified if data are treated ordinally, but necessarily misspecified (primarily regarding factor loadings) if data are treated continuously. However, the finding in [14] is trivial and without any consequence for actual data. In [15], the authors showed that treating ordinal items in an ordinal FA can lead to biased estimates while treating them continuously will not (i.e., one can write $\Sigma =\Lambda \Phi \Lambda^{⊤}+\Psi$ for the Pearson correlation matrix Σ). I simply assume that the FA model holds for the Pearson correlation matrix, not for the polychoric correlations. Hence, the two methods (i.e., ordinal and continuous treatment of the data) rely on different assumptions, and researchers have to decide which of them is more plausible. However, in my opinion, general statements about “biased parameter estimates” are unscientific and should not be part of standards for research practice.

Moreover, one can only question whether the “assumption of equidistant categories” in items is reasonable. However, researchers must be aware that ordinal FA defines distances between item categories on an empirical basis. That is, the observed frequency distribution defines how categories should be scored. More critically, the definition of distances between item categories additionally depends on the assumption of an underlying latent normally distributed variable [16]. This normality assumption is entirely arbitrary [17], and any other distribution of the underlying latent variable could be defended. Relying on the assumption of equidistant categories at least has a clear interpretation, while the treatment of the distances of item categories in ordinal FA is entirely atheoretical because it is defined by the sample distribution.

Overall, it would be more fair to say that either the continuous or the ordinal FA imposes incorrect distributional assumptions. The latent normality assumption in ordinal FA is, unfortunately, often taken for granted, but it can be tested [18]. More flexible distributions could be identified from data (e.g., [19]). However, such a data-driven approach remains atheoretical, and it is questionable whether measurement models with more flexibly estimated distributions would be more appropriate and provide more valid results.

## 4. Estimation Methods in Exploratory and Confirmatory Analysis

In CFA, ConPsy generally regards maximum likelihood (ML) estimation methods for continuous data “as superior [to unweighted least squares (ULS)] in terms of their robustness” (see also [20,21]). I think that decisions among different estimators require some elaboration. First, the data distribution could deviate from multivariate normality. In this case, FA can still be applied because it is just a representation of the covariance matrix. However, ML estimation might not be the most efficient estimation method in skewed or heavy-tailed distributions. Hence, robustness is understood in terms of the data distribution in the sample. Second, the CFA model can be misspecified. For example, there could be unmodelled residual error correlations. In this case, robustness means that model deviations should impact estimated model parameters as little as possible. This property of model robustness [22,23] must be clearly distinguished from robust estimation regarding distributional misspecification of the data. The model-robustness property should only be considered if model errors are sparsely distributed (i.e., only a few of the modeled covariance matrix entries in the FA are incorrectly specified). If model errors are densely and rather unsystematically distributed, ULS might be preferred over ML estimation [24].

## 5. Reliability

ConPsy distinguishes three approaches to reliability assessment: internal consistency measures, test–retest reliability, and inter-rater reliability. I will only focus on reliability assessments based on internal consistency in this section. Cronbach’s alpha [25] is likely the most frequently used reliability measure. ConPsy critically remarks that

“Cronbach’s alpha assumes that measurement error is random and is influenced by the sample used. It also assumes unidimensionality and equal factor loadings across indicators, which may not always be justified for latent constructs.”

Unfortunately, I disagree with all parts of this quote. In [25], no dimensional assumption was imposed on a test when the reliability coefficient was rooted in classical test theory (CTT). Items used in an instrument are defined as exchangeable and are representative of the domain of items. Cronbach’s last paper [26] explicitly states that only the representativity assumption is crucial for deriving the reliability coefficient (see also [27]). This is a design-based perspective on reliability [28,29]. Alternatively, researchers can define model-based reliability measures based on an FA [30]. In this case, reliability is defined based on model parameters obtained from a factor model. Then, it is argued that Cronbach’s alpha provides biased reliability (see, e.g., [31]). However, this reasoning critically relies on the model-based definition of reliability. I reiterate that the derivation of Cronbach’s alpha does not employ this assumption. As pointed out by an anonymous reviewer, a larger value of alpha does not automatically imply a larger degree of unidimensionality [32].

McDonald’s omega [33] is the most frequently employed reliability measure based on FA. ConPsy states that

“Reliability measures have also been developed within the framework of latent variable modelling, with McDonald’s omega proposed as a more suitable measure for reliability in studies using structural equation modelling (SEM) compared to Cronbach’s alpha [...]. Omega is based on the congeneric model, which allows for non-equivalent factor loadings across items.”

As I argued above, I think it is unsuitable to prefer omega over alpha based on a model-based reasoning because both coefficients are derived under different assumptions. Researchers should not be blamed for using alpha if they want to determine reliability on the exchangeability assumption of items.

The controversies regarding the appropriate reliability measure are likely still alive because a lot of researchers believe that CTT relies on an FA model with equal loadings. In my view, this is an incorrect description. CTT has fewer assumptions than FA because it only relies on exchangeability. In this regard, one can argue that FA treats items as fixed while CTT treats items as random (see [34]).

Furthermore, I would like to emphasize that alpha and omega are reliability measures of the unweighted sum score. In many articles, conclusions among latent constructs are obtained by operationalizing them as latent variables in a structural equation model (SEM). Critically, the reliability of the factors implicitly used in SEMs does not refer to the reliability of the unweighted sum score. Hence, omega would be an incorrect choice as a reliability measure if something should be stated about the reliability (i.e., internal consistency) of a factor in a CFA or SEM. Hancock’s *H* would be a more appropriate reliability measure in this case [35].

I think that ConPsy should also alert researchers using an adequate factor-based reliability omega when items are treated in an ordinal FA. In my experience, researchers frequently compute omega from the FA output based on the polychoric correlation matrix. However, it has been pointed out in [36] that the omega computation for ordinal items must also take the location of item thresholds into account. Ironically, a reasonable reliability omega frequently results if it is (incorrectly, according to ConPsy) based on the FA for continuous items.

Importantly, reliability statistics can and should be defined for measures that fit an EFA model with multiple factors [30]. In this perspective, the set of chosen items in the instrument represents a domain, and the appropriate selection of items in the instrument is ensured by representativity arguments (e.g., by expert interviews). The EFA is used to represent the covariance structure, and only the item-specific variance components in this model are treated as unreliable, while the explained variance by the factors represents true variance. In this domain sampling approach to reliability [37,38,39,40,41,42,43], it is important to emphasize that the factors in the EFA must not be interpreted; they are only used in the statistical model.

## 6. Measurement Invariance

Measurement invariance (MI) is frequently “ceremonially performed” [44], in particular in psychological research. In a nutshell, MI regarding a discrete covariate (i.e., groups) holds if factor loadings and item intercepts do not possess group-specific parameters. This means that all observed group differences in items must be represented as group differences in group-specific factor means and factor variances. In fact, ConPsy also clearly belongs to this kind of researcher who believe that rigorous measurement can only be established if MI is tested:

“Measurement invariance is a crucial property of a measurement scale that ensures bias due to exogenous factors does not affect the measurement. If measurement invariance is not established, interpreting score differences is theoretically not advised.”

Among others, I strongly disagree with these claims [45]. MI is neither necessary nor sufficient for measurement validity [45,46,47]. It is not sufficient because exogenous factors of covariates that homogeneously affect items remain undiscovered in the assessment of MI. It is not necessary because I think that the heterogeneous functioning of items across groups is not a bug but an in-built measurement feature [48,49]. For example, I do not know why it should be reasonable that six chosen self-concept items are not allowed to function differently for men and women. Can we still compare men and women regarding self-concept if MI is violated? ConPsy notes that

“[...] establishing measurement invariance in psychometric scales is crucial for meaningful comparisons of factor and total scores (structural invariance) between different groups or conditions”

Hence, according to ConPsy, either full MI or partial MI (i.e., a few group-specific item parameters are allowed) seems required “to make meaningful comparisons between groups” [1]. I do not think that it is a useful recommendation for researchers. A violation of MI in the example of the test with six self-concept items implies that any subset of these items would result in a gender difference that differs from the analysis based on all items. To me, this does not cause any issue if the test is held fixed; that is, the inference is made exactly for these six chosen self-concept items.

It has been pointed out that violations of MI can be attributed to true group differences because a multidimensional construct is intended to be measured. In this case, measurement noninvariance is construct-relevant [50,51,52]. If noninvariant items receive group-specific item parameters in a partial invariance FA model, these items would be practically removed from group comparisons [53]. Hence, the statistical model practically changes the substantive question of interest [45]. It is the careful examination of noninvariant items that could make the MI assessment a beneficial psychometric analysis. In this sense, the assessment of noninvariance must focus on the level of items [54] and not on the mechanistic global model comparisons. I reiterate by stressing that noninvariant items should only be removed from comparisons if researchers provide reasons for the noninvariance that are construct-irrelevant.

It should be emphasized that the statistical test of MI is clearly defined. However, the practical implementation of the partial invariance approach is entirely arbitrary. Under violation of MI, researchers can defend any assumption for defining group differences [55]. The modeling alternatives in the absence of (full) MI cannot be tested against each other. Furthermore, I think that the situation of partial invariance, in which only a few group-specific item parameters differ from zero while the majority of parameters have a value of zero, will be as rare as unicorns in applied research.

Ref. [56] complains about the prohibitive tone of proponents of the MI ceremonies. It is argued that “the amount of non-invariance required to actually throw substantive results into question is far from clear and, as noted above, often is evaluated on the basis of mysterious and seemingly arbitrary benchmark” [56], concluding that “[...] it means if you do not have strict MI, your mean differences do not mean anything. So, you are prohibited to look at them – an attitude that strikes me as, how shall I put this, anti-scientific” [56]. The assessment of measurement quality does not become (more) scientific if only partial MI must apply. The discussion on how to handle deviations of MI is also conceptually relevant, although several studies demonstrated only minor differences in the consequences of different analysis strategies [57,58].

## 7. Validity

Unsurprisingly, it is more difficult to come up with standards for the assessment of validity. ConPsy states that

“In psychometric evaluation, the first step is to assess the dimensionality of a tool, as reliability is to be assessed for each dimension separately. Validity on the other hand is the last to be assessed as a tool cannot be valid unless shown reliable.”

I strongly disagree with the last sentence. In contrast to ConPsy, I think that researchers are often confronted with the reliability–validity paradox [59]. That is, an increase in reliability can lower the validity and the other way around. Such a situation frequently occurs if the instruments consist of multiple facets for which reliability differs [60]. It seems that ConPsy believes that all measurement instruments should be constructed so that all items load only on one dimension. I think this constitutes an unnecessarily restrictive measurement device. Note that the reliability–validity paradox can also be formulated in the sampling model of validity in generalizability theory [61].

If items are selected for measurement, internal item validity (factor loading on a single dimension) must be distinguished from (external) item validity, in which heterogeneous functioning of items regarding an external variable is allowed. MI would be clearly violated in the latter case. However, from a validity perspective, researchers could, for example, choose the items that have the largest instructional sensitivity in an educational intervention [62,63]. I think such a test construction principle would result in the highest validity, and researchers should not start with the assessment of the reliability in the first step.

## 8. Quantifying Model Error in Factor Analysis in Increased Standard Errors

The final analysis models of interest that will be published in an article will likely be misspecified to some extent. Researchers that employ CFAs or SEMs either try to play down model misspecification (i.e., model error) by relying on choosing appropriate effect sizes of model fit to hide misfit or to conduct model modifications by including additional model parameters. In the latter case, researchers typically include residual correlations, cross-loadings, or group-specific item parameters in FA models relying on partial invariance. Importantly, standard errors reported in the final model do not reflect the sampling uncertainty involved in the analysis steps conducted for model refinement. Furthermore, I would also argue that the meaning of factor variables substantially differs whether cross-loadings are allowed or not. It seems questionable to me that a statistical model (i.e., the FA model) suggests including cross-loadings, which essentially changes statements about the measured constructs.

I tend to prefer a different kind of modeling strategy. The CFA model with a simple loading structure and the multiple-group CFA model assuming MI are measurement ideals. Data will typically not fit the imposed FA models well. However, the whole instrument (i.e., the set of items) correctly measures the construct (and, hence, the FA model) “on average”. Therefore, there are unsystematically distributed model errors in the imposed CFA model. However, looking at the largest model deviations (i.e., using modification indices) does not make sense because all model errors will differ from zero. I would like to see the approach of [64] being widely implemented in SEM/CFA software. In this approach, the extent of model error is reflected in increased standard errors [24,64,65,66,67]. This would be a more honest strategy to acknowledge the extent of SEM/CFA model misspecification.

## 9. Are Factor Models Needed at All for the Evaluation of Measurement Instruments?

It is striking that FA models cover a large part of the ConPsy psychometric checklist. Obviously, this class of statistical models seems to play a central role in assessing measurement quality. However, one could ask whether researchers need factor models at all to assess measurement quality. In the physical sciences, it would seem awkward to demonstrate the adequacy of a measurement instrument by an appropriate fit of measurement observations to a statistical model, such as the FA model. In the social sciences, it seems that the application of FA models compensates for the absence or vagueness of substantive theory between the (latent) construct and measurement situations (i.e., items). That is, a factor in a CFA is unjustifiably interpreted as an operationalization of a latent construct [68]. I simply do not see why the appropriateness of a statistical model should give an insight into whether successful measurement has been established.

Nunnally and Bernstein [69] (p. 316) argue that factor analysis at the level of items is not encouraged and is unnecessary if the item domain is well defined by content experts. They state that “ordinary approaches to factoring items [...] are almost guaranteed to lead to spurious results”. I fully agree with this view. Statistically extracted factors in an EFA or a CFA should not be equated with a proper definition of measurement.

That being said, I do not argue that EFAs or CFAs cannot be informative statistical tools. Likely, they could be useful to detect some irregularities in items. Moreover, they can effectively summarize items in a factor variable which is used in subsequent analysis. Furthermore, an SEM or a CFA has an in-built adjustment for unreliability. However, it can be questioned whether the reliability that is implied by specifying a factor variable for a set of items will provide an appropriate reliability. Constructs are typically multidimensional; thus, I can only think of a few instances in which I would prefer an SEM with multiple factor variables and its measurement models over a path model that replaces factor variables with a weighted sum of their items. In the latter models, corrections for the (appropriate) unreliability can easily be included, and the techniques discussed in the rich literature of measurement error models [70,71,72] can be employed.

## 10. To Check or Not to Check?

I think that it is notoriously difficult to propose standards in a research area. There will always be some kind of disagreement among researchers about what they see as important standards in research practice. I believe that it is at least helpful in some instances that different researchers conclude that they agree to disagree with each other. Hence, some researchers will likely check some standards while others will not.

## Abbreviations

The following abbreviations are used in this manuscript:

CFA	confirmatory factor analysis
ConPsy	contemporary psychometrics
CTT	classical test theory
EFA	exploratory factor analysis
FA	factor analysis
MI	measurement invariance
ML	maximum likelihood
SEM	structural equation model
STV	subjects-to-variables
ULS	unweighted least squares
